# Astrocyte-Secreted Matricellular Proteins in CNS Remodelling during Development and Disease

**DOI:** 10.1155/2014/321209

**Published:** 2014-01-16

**Authors:** Emma V. Jones, David S. Bouvier

**Affiliations:** Centre for Research in Neuroscience, Department of Neurology and Neurosurgery, The Research Institute of the McGill University Health Centre, Montreal General Hospital, Montreal, QC, Canada H3G 1A4

## Abstract

Matricellular proteins are secreted, nonstructural proteins that regulate the extracellular matrix (ECM) and interactions between cells through modulation of growth factor signaling, cell adhesion, migration, and proliferation. Despite being well described in the context of nonneuronal tissues, recent studies have revealed that these molecules may also play instrumental roles in central nervous system (CNS) development and diseases. In this minireview, we discuss the matricellular protein families SPARC (secreted protein acidic and rich in cysteine), Hevin/SC1 (SPARC-like 1), TN-C (Tenascin C), TSP (Thrombospondin), and CCN (CYR61/CTGF/NOV), which are secreted by astrocytes during development. These proteins exhibit a reduced expression in adult CNS but are upregulated in reactive astrocytes following injury or disease, where they are well placed to modulate the repair processes such as tissue remodeling, axon regeneration, glial scar formation, angiogenesis, and rewiring of neural circuitry. Conversely, their reexpression in reactive astrocytes may also lead to detrimental effects and promote the progression of neurodegenerative diseases.

## 1. Introduction

Astrocytes secrete numerous factors and active molecules [1–4], which modulate synapse development, neuronal activity, and plasticity during development and in the mature brain [5–10]. In particular, immature astrocytes produce and secrete many types of proteins that allow them to remodel the extracellular matrix (ECM) surrounding neurons and synapses [11]. In addition, astrocytes have the ability to react to any kind of insult or change in their environment, physical, viral, or chronic disease, and work side by side with microglia, the immune cells of the brain, in order to contain and to repair the brain from injuries [12, 13]. Astrocyte reactivity is associated with striking changes in gene expression and morphology [14, 15]. It has been demonstrated that reactive astrocytes revert to a partially immature molecular profile [16], which allow them to reexpress a variety of factors/proteins required for tissue remodelling around injury sites [13]. Some of these proteins have direct roles on modulation of ECM and cell to cell interactions. One mechanism used by reactive astrocytes to reshape their microenvironment is through the secretion of matricellular proteins.

Matricellular proteins are a family of structurally unrelated proteins that are secreted into the extracellular space. They act as nonstructural regulators of the ECM and cell-matrix interactions through modulation of growth factor signaling, cytokines, hormones, and proteases [17]. In general, matricellular proteins are highly expressed during development and present at a lower level during adulthood. However, their expression is upregulated following injury or disease. Interestingly, mice with homozygous-null mutations for this family of molecules do not usually exhibit gross abnormalities and generally have subtle phenotypes until challenged [18]. In contrast with other extracellular matrix proteins, matricellular proteins have been shown to have deadhesive activity, which likely contributes to their role in tissue remodeling following injury or in disease states. The matricellular protein family comprises many members to date (recently reviewed in [19]). Although widely studied in nonneuronal tissues, their roles in the CNS are not well understood. This minireview will focus on the expression and function of matricellular proteins secreted from astrocytes of the CNS, both in development and in the context of glial reactivity and disease, namely, SPARC (secreted protein acidic and rich in cysteine, also known as osteonectin/BM-40), Hevin (also known as SPARC-like 1 or SC1), Tenascin C (TN-C), Thrombospondins (TSP), and the CCN (CYR61/CTGF/NOV) family.

## 2. SPARC and Hevin/SC1

SPARC/osteonectin/BM-40 (now referred to as SPARC) and SC1/Hevin, first discovered two decades ago, are part of a larger family of SPARC family proteins, which share a common E-F hand calcium binding (EC) domain [20–23].

Of the SPARC family proteins, Hevin has the greatest homology to SPARC and both proteins have three main domains; an N-terminal acidic calcium-binding domain, a central cysteine-rich follistatin domain, and the C-terminal EC domain [24] which confers collagen-binding [25–27]. SPARC has been shown to modulate several growth factor signaling cascades (i.e., VEGF (vascular endothelial growth factor), PDGF (platelet-derived growth factor), FGF2 (fibroblast growth factor-2), and TGF*β* (transforming growth factor beta) and can regulate integrin-mediated adhesion (reviewed in [28]). Mice containing targeted deletions of either SPARC or Hevin are viable and fertile and generally exhibit relatively mild phenotypes until revealed by injury or challenge [20, 29, 30]. However, recent studies of SPARC and Hevin knock-out animals have revealed important roles for these molecules in CNS development [9, 31–33].

During embryonic development, SPARC and Hevin have somewhat overlapping expression profiles and are found in radial glia and the developing vasculature (recently described in detail in [34, 35]). Indeed, Hevin was shown to be important for appropriate termination of radial glia-guided neuronal migration and proper lamination of the cerebral cortex [32].

During the first few weeks of postnatal development, SPARC and Hevin are highly expressed in astrocytes [9, 31], with Hevin also being present in neurons [36]. Secretion of SPARC and Hevin from astrocytes has been shown to play an important role during synapse development (recently reviewed in [5, 11]). Kucukdereli and colleagues [31] demonstrated that astrocyte-secreted Hevin promotes the formation of excitatory synapses in the superior colliculus. They proposed that SPARC may compete with Hevin to regulate synapse development, since SPARC was found to antagonize the synaptogenic effects of Hevin on cultured RGC neurons. In contrast, morphological synapse development in hippocampal neurons cultured with SPARC null astrocytes versus wild type astrocytes was similar [9]. However, synapses in SPARC null cultures were found to have a greater number of AMPA receptors and an increase in synaptic strength, which was rescued through application of recombinant SPARC. Regulation of AMPA receptors by SPARC required *β*3-integrin signaling [9]. A recent study has shown that these synaptic changes can lead to alterations in hippocampal-related behaviours in the adult. Campolongo and colleagues demonstrated that SPARC knock-out mice exhibited an increase in anxiety and antidepressant-like behaviours [29].

Taken together, these studies suggest that SPARC may play an important role in excitatory synapse development by preventing premature maturation of synapses to facilitate proper formation of neural circuitry. How Hevin and SPARC might interact to exert their effects on synapses is not yet understood.

### 2.1. SPARC and Hevin Are Upregulated in Reactive Astrogliosis

In the mature CNS, the expression of SPARC and Hevin is reduced, and, in the case of SPARC, its expression is confined to radial-like glia cells such as Bergmann glia and Muller glia, resting microglia at low levels in astrocytes [35, 37]. In contrast, Hevin expression in the adult brain is restricted to mature astrocytes and neurons and is not present in microglia [34, 38]. However, upon injury or disease, the expression of both proteins is upregulated in reactive astrocytes [39, 40]. Hevin is found in the cell bodies of reactive, dividing astrocytes and in hypertrophied processes surrounding neurons and blood vessels following status epilepticus in rat [41]. Hevin is also highly expressed in reactive glia following lesion injury [42, 43] and ischemic stroke [44], where Hevin expression was shown to be upregulated in astrocytes surrounding the lesion at 1 day after stroke and was sustained for up to a week. Similarly, SPARC has been shown to be upregulated in reactive astrocytes following injury [39] and stroke [45]. Interestingly, SPARC upregulation occurred in a slow sustained manner in both of these studies, suggesting that SPARC produced from reactive astrocytes may play a role in later, rather than immediate, processes of recovery and repair. 

In the human brain, SPARC is expressed in reactive human astrocytes proximal to brain tumours [46] and was shown to be increased in cases of human epilepsy [47]. SPARC is expressed in several types of brain tumours, and its expression in astrocytomas and gliomas is generally associated with increased invasion, angiogenesis, and a negative prognosis [48, 49]. The role of Hevin in cancer is likely complex: its expression has been shown to be correlated with invasive gliomas [50] yet it is highly downregulated in other malignancies and has been described as a tumour suppressor gene [24, 51].

### 2.2. Cleavage of SPARC and Hevin and the Generation of Functional Protein Fragments

The role of Hevin and SPARC in cancer, brain injury, and disease is likely dependent on the tissue type and other molecules in the ECM environment, such as the presence of proteases. Both SPARC and Hevin have been shown to be cleaved by members of the matrix metalloproteinases (MMP) family to release functional protein fragments. Hevin can be cleaved by MMP-3 and ADAMTS4 (a disintegrin and metalloproteinase with Thrombospondin motifs) to release a shorter, C-terminal fragment that is highly homologous to SPARC, known as “SPARC-like fragment” (SLF) [38, 52]. Interestingly, the SLF was shown to oppose the synaptogenic action of full length Hevin [31, 52] and was found associated with SPARC in neovasculature of gliomas [52]. Furthermore, it is possible that SPARC may play a role in the proteolysis of Hevin, since SPARC has been shown to induce the expression of MMPs [53, 54]. SPARC is regulated by MMP proteolysis and is cleaved by MMP-3 *in vitro* to produce protein fragments that have differential effects on cell proliferation and migration [55]. Peptides encompassing different domains of SPARC (such as would be released by MMP cleavage) have been shown to have potent effects. For example, “peptide 2.3,” which contains part of the follistatin-like domain of SPARC, regulates SPARC-*β*-integrin interactions in nonneuronal cells [56], can stimulate angiogenesis activity [57], and rescues *β*-integrin and AMPA receptor overaccumulation at synapses in SPARC null hippocampal cultures [9].

MMPs and their inhibitors, TIMPs (tissue inhibitors of metalloproteinases), are upregulated in reactive astrocytes in a range of disease contexts such as following ischemic stroke or in Alzheimer's disease (AD), where they are thought to have an important role (reviewed in [58, 59]). In ischemic stroke or injury, MMP expression has been shown to have a detrimental role in the initial stages [60], but it is protective in the later recovery phases where it is important for promoting angiogenesis of new blood vessels and restoration of the blood brain barrier [61]. The role of MMPs in angiogenesis is thought to involve VEGF signaling [58], which has also been shown to be regulated by SPARC (reviewed in [28]). Given that MMP can regulate SPARC and vice versa, it would be interesting to determine whether MMP and SPARC could coordinate regulation of tissue remodeling and angiogenesis following injury to the CNS.

Currently, the role of SPARC and Hevin in the CNS has been mostly studied in the context of development. One interesting question is whether these proteins will have similar functions in the adult CNS following injury or disease. For example, are SPARC and Hevin involved in *de novo* synapse formation and circuit rewiring following CNS injury? Does their function require proteolysis? Answers to these questions will help provide insight into the functions of SPARC and Hevin produced from reactive astrocytes and their role in modulating the ECM environment.

## 3. Tenascin C

Tenascin C (or TN-C with C for cytotactin) is a secreted extracellular matrix glycoprotein that is part of a family of three homologs along with Tenascin R and Tenascin X. TN-C was discovered in the early 1980s and has carried numerous names like myotendinous antigen, glioma mesenchymal extracellular matrix, hexabrachion, tenascin, Jl-200/220, and cytotactin [62]. TN-C is composed of several distinct domains containing epidermal growth factor-like repeats, fibronectin type III repeats, and a segment of great homology with the *α* and *β* chains of fibrinogen [62, 63]. It is transiently expressed by neural and nonneural cells during development and plays a role in the ECM remodelling during tissue repair. During brain development, the expression of TN-C by radial glia and subpopulations of astrocytes has been shown to affect multiple processes such as cell migration and proliferation, axonal guidance, and synaptic plasticity.

### 3.1. Astrocyte-Secreted TN-C Is a Key Molecule in the Establishment of Neuronal Circuitry

TN-C was first described as a protein produced and secreted *in vitro* by astrocytes in culture [64, 65]. It was demonstrated that its expression was limited to specific astrocyte cell lines or subpopulations during CNS development [66, 67]. Paradoxically, TN-C was considered both as a repulsive substrate for neuronal and astrocytic growth [68, 69] and as a permissive one, by providing axonal guidance cues [66, 67, 70, 71]. Depending on the context, such as the expression of other ECM molecules, TN-C can have the opposite differential effects on neuronal growth. TN-C is also known to influence astrocyte proliferation and induces process elongation through an autocrine/paracrine mechanism [63]. In addition, TN-C has been shown to act through *β*1 and *α*9 integrin-dependent cell adhesion [69, 72] and to interact physically with the neuronal GPI—membrane-anchored adhesion glycoprotein F3/contactin of the Ig superfamily [71].

TN-C is expressed *in vivo* in various brain regions and exhibits a precise temporal and spatial distribution. In the somatosensory cortex of rodents, TN-C was detected by immunohistochemistry in populations of astrocytes delineating the boundaries of whisker barrel fields in the early postnatal development (P1–P7) [73]. By P9, TN-C expression in barrels has almost disappeared. TN-C was also reported to be transiently expressed by cortical radial glia cells [74] and by astrocytes of the optic nerves [75]. In the cerebellar cortex, TN-C was found to be highly expressed in the first four postnatal weeks before being downregulated [75]. Using *in situ* hybridization and electron microscopy, the authors showed that TN-C was mainly produced and secreted by astrocytes and epithelial cells and was only detected at the cellular surface of neurons. However, in the developing hippocampus, TN-C not only is expressed by radial glia cells and immature astrocytes but also by a subset of neurons of the stratum oriens [76]. In the spinal cord, TN-C is synthesized by a subset of gliogenic precursors in the late phase of embryogenesis and has been shown to influence proliferation and migration of subpopulation of astrocytes [77].

Surprisingly, TN-C null mice do not show a major phenotype or alteration in the gross histoarchitecture of the CNS but do display subtle morphological alterations in some subtypes of neurons [78, 79]. In addition, TN-C null mice exhibited an enhanced proliferation but a delayed migration of immature astrocytes toward the ventral spinal cord white matter [77]. Furthermore, it has been shown that the number of cells is changed in the cerebral cortex of TN-C null mice when compared to the WT animals, with an abnormally high neuronal density and increased astrogliosis but low density of parvalbumin-positive interneurons and reduced ratio of oligodendrocytes [79]. Evers and colleagues observed plasticity impairments in the hippocampus of one-month-old TN-C null mice. Indeed, intertheta burst stimulation-(TBS-) induced LTP (long term potentiation) were shown to be significantly reduced and LTD (long term depression) abolished at Schaffer collateral—CA1 synapses whereas LTP in the dentate gyrus or CA3 were described as normal [78]. The authors demonstrated a causal effect between the deficiency in TN-C and an impairment of the L-type Ca2^+^ channel-dependent forms of synaptic plasticity in the CA1 that could be mediated by interactions with integrins or proteoglycans. In addition, TN-C −/− mice trained in the step-down avoidance test also showed clear deficits in contextual memory [80]. Interestingly, different domains of TN-C have distinct functional properties. Indeed, the intrahippocampal injections in wild type animals of the recombinant fragment of TN-C containing the fibronectin type III repeats 6–8, but not 3–5, blocked memory formation and suggests that TN-C can directly modulate synaptic plasticity [80]. Therefore, the reexpression of TN-C by reactive astrocytes during injuries or brain diseases could impact the processes of cell proliferation, migration, and synaptic plasticity.

### 3.2. TN-C in Astrogliosis and Brain Diseases

Tenascin C is poorly expressed in adult brain. However, it has been observed to be upregulated at injury sites in correlation with astrocyte reactivity and glial scar formation. Indeed, stab wound in cerebellar and cerebral cortices structures have been shown, by *in situ* hybridization and immunohistochemistry, to enhance the production of TN-C in a discrete population of GFAP-(glial fibrillary acidic protein) positive astrocytes proximal to the injury site [81]. TN-C is also intensely expressed by astrocytes in culture in scratch wound assays [82]. Interestingly, TN-C could be involved in the maintenance of astrogliosis surrounding site of severe injuries. Indeed, stab wound assays in cerebral cortex revealed that GFAP expression, which is usually correlated with severity of reactivity, was significantly weaker in TN-C null versus wild type mice, one week after the stab [83]. However, in the same study, IgG leakage persisted much longer in TN-C-deficient mice and RNA levels of proinflammatory cytokines TNF*α* (tumour necrosis factor alpha), IL-6 (Interleukin-6), and IL-1*β* levels were higher [83]. Thus, the production of TN-C may influence blood-brain barrier (BBB) repair and the regulation of inflammatory cytokine levels either directly or indirectly through modulation of BBB integrity. In addition, TN-C production in astrocytes can be modulated by inflammatory cytokines and growth factors. Indeed, astrocytes increased their production of TN-C when cultured in presence of activated macrophage- and microglial-conditioned media [84]. Smith and colleagues have also demonstrated that the synergic action of both TGF-*β*1 and basic fibroblast growth factor (bFGF), both factors known to be upregulated in injury, stimulates the expression of TN-C [84].

Interestingly, TN-C upregulation has been described in numerous neurodegenerative diseases. TN-C expression is induced in the hippocampi of both epileptic rat brains [85, 86] and human patients with temporal lobe epilepsy (TLE) [87]. In brains of TLE patients, the regions exhibiting a diffuse and elevated expression of TN-C were also characterized by an extended area of reactive gliosis and synaptic reorganization. In addition, TN-C was recently reported as a plasma biomarker for neurodegenerative diseases, as levels were found to be significantly increased in blood of Alzheimer's disease (AD), mild cognitive impairments patients [88, 89], and in the amniotic fluid of Down syndrome-affected pregnancies [90]. In addition, TN-C level was shown to be upregulated in the AD-like mouse model CRND8 when compared to the wild type littermates [91]. Furthermore, TN-C deficiency in transgenic CRND8 mice provoked a reduction of *β* and *γ* secretase activity, A*β* oligomerization, plaque load, and synaptic impairments. In addition, loss of TN-C in CRND8 Tg mice enhanced production of anti-inflammatory cytokines and reduced proinflammatory cytokines [91]. This contrasts with the effect of TN-C deficiency on inflammation levels in the context of physical injury [83]. Therefore, TN-C may act as a promoter or inhibitor of the inflammation depending on the type of insult. In support of this, TN-C has also been demonstrated to induce production of proinflammatory cytokines via activation of Toll-like receptor 4 (TLR4) in nonneuronal tissue [92]. Interestingly, TLR4 is known to be expressed at the surface of astrocytes [93, 94] and microglia [95] and is directly implicated in the induction of inflammation in various neurodegenerative diseases [95–97].

In conclusion, the upregulation of TN-C by reactive astrocytes in response to an insult to the brain is context specific and may be a key factor in multiple processes such as the maintenance of astrocyte reactivity, BBB repair, and the potentiation of inflammatory processes. It could also directly affect neuronal plasticity and lead to memory impairments.

## 4. Thrombospondins 

There are five known thrombospondins (TSP, also known as THBS) in vertebrates, which can be classified into two groups: the trimeric TSP1 and -2 and the pentameric TSP3, -4, and 5. All of the TSPs have epidermal growth factor-(EGF-) like repeats followed by calcium-binding type 3 repeats, and all share a highly conserved C-terminal region [98]. Thrombospondins are secreted molecules and interact with structural components of the ECM as well as proteases, cytokines, and growth factors to modulate cell signaling, adhesion, migration, and other cellular processes [99]. TSPs have many known binding partners, including integrins, neuroligins, Reelin receptors ApoER and VLDLR (very low density lipoprotein receptor), the calcium channel subunit *α*2*δ*-1, and several growth factor ligands and their receptors (summarized in [100, 101]).

The different TSPs are expressed throughout the organism at various stages of mammalian development [98]. In the CNS, TSPs 1 and 2 are expressed in cultured and developing astrocytes, with expression peaking during the first postnatal week in mice [102, 103]. In addition, high levels of TSP1 can be found in cultured human astrocytes [104, 105]. Astrocyte-secreted TSP1/2 has been shown to be important for the formation of excitatory synapses *in vitro* and *in vivo* ([102, 106], recently reviewed extensively in [11, 101]). Briefly, TSP treatment of cultured neurons led to an increase in the number of synapses [102] and rate of synaptogenesis [107]. Synapses induced by TSPs are morphologically normal but postsynaptically silent, indicating that TSP is important for the initiation of synapse formation, but other secreted factors are required for completion of synapse maturation [5, 9], reviewed in [108].

### 4.1. Expression of TSPs in the Mature CNS and Following Injury

In mature astrocytes, the expression of TSP1/2 is decreased, although a low level is maintained throughout the brain and is particularly concentrated in areas of neurogenesis in the adult (i.e., subventricular zone (SVZ) and subgranular zone (SGZ)) [102, 109–111]. Indeed, loss of TSP1 was associated with a decrease in the number and distribution of SVZ neural precursors entering the olfactory bulb from the rostral migratory stream [112]. In contrast to TSP1/2, expression of TSP4 is low during development but present in adulthood, where it is expressed in spinal cord astrocytes [113] and mature forebrain astrocytes [114] and was found localized to synapses at neuromuscular junctions in the peripheral nervous system [115].

Similar to the other astrocytic matricellular proteins, TSPs are upregulated in reactive astrocytes following ischemic [116, 117] and mechanical [118] injury. It is likely that several signaling pathways can stimulate TSP production in reactive astrocytes. For example, in an *in vivo *trauma model, TSP1 expression was induced by extracellular ATP (Adenosine triphosphate) via P2Y purinergic receptors and ERK (extracellular signal-regulated kinase)/p38 MAPK (mitogen-activated protein kinase) signaling [119]. Additionally, TSP1 expression was activated by collagen-stimulated integrin signaling in reactive astrocytes following brain injury [120].

Following stroke, TSP1 and -2 display a differential temporal expression pattern; TSP1 was shown to be upregulated in the penumbra (the region surrounding the lesion core) within 3 days, whereas changes in TSP2 expression were delayed and levels were only increased at one week after stroke [116, 117]. Interestingly, the peak in expression of TSP1 and TSP2 was inversely correlated with angiogenic activity in the penumbra, consistent with a role for TSP1/2 in regulation of angiogenesis [121]. Conversely, Liauw and colleagues [116] reported that loss of TSP1/2 did not affect blood vessel density after stroke. Furthermore, they suggested that TSPs play a beneficial role in synaptic recovery, since TSP1/2 null mice exhibited a reduction in synapse number and axonal sprouting (and consequent motor function) when compared to wild type mice. This is particularly interesting considering that several studies have shown that TSPs can promote neurite outgrowth of cultured neurons *in vitro *[122–125] and is expressed around regenerating axons following injury *in vivo* [126, 127]. In addition, TSPs have been shown to induce dendritic spine formation [105] and are important for synaptogenesis ([102, 107] discussed in text above). In support of this, TSP expression is downregulated in diseases characterized by synaptic loss or abnormal synapse formation, such as in AD [128] and Down syndrome [105]. Taken together, upregulation of TSP1/2 expression in reactive astrocytes may contribute to tissue recovery following injury by promoting *de novo* synapse formation and rewiring of neural circuitry.

One key question in the field is where reactive astrocytes come from? Do they develop from local mature astrocytes or do they originate from neurogenic niches and migrate to the site of injury? It is possible that both of these processes occur depending on injury context [129]. In addition, it is thought that the molecular profile of reactive astrocytes overlaps with that of immature astrocytes or radial glial cells [16]. Recently, recruitment of newborn astrocytes and neurons from the SVZ was shown to play an important role following ischemic injury and stroke [130, 131]. Importantly, TSP4 was shown to be upregulated in SVZ-derived astrocytes surrounding lesions induced by photothrombotic injury and was required for injury-induced astrogenesis of neural stem cells, which was mediated through Notch signaling. Loss of TSP4 led to an increase in hemorrhaging and changes in glial scar formation [131]. In addition, expression of TSP4 was increased following peripheral nerve injury in spinal cord astrocytes, which morphologically resembled immature astrocytes [113]. Further study of TSPs may assist in the understanding of astrocyte heterogeneity and the process of astrogliosis.

## 5. CCN Family

The matricellular protein family CCN, which stands for CYR61/CTGF/NOV, are a family of six homolog members containing CYR61/CCN1 (cysteine-rich 61), CTGF/CCN2 (connective tissue growth factor), NOV/CCN3 (nephroblastoma overexpressed), and WISP-1/CCN4, WISP-2/CCN5, and WISP-3/CCN6 (Wnt-inducible-secreted proteins). All CCN proteins have a modular structure, which consists of four conserved cysteine rich-domains, with sequence homology to the insulin-like growth factor-binding proteins (IGFBP), the von Willebrand factor C (VWC) domain, Thrombospondin type 1 repeat (TSR), and a C-terminal domain with a cysteine-knot motif. CCN proteins can bind to integrin receptors and coreceptors such as heparan sulfate proteoglycans (HSPGs), low-density lipoprotein receptors-related proteins (LPRs), and TRKA [132]. They have been reported to participate in multiple functions in non-CNS organs, such as in migration, proliferation, and apoptosis (reviewed in [132]). They are upregulated in chronic inflammatory diseases, where they are suggested to be important players in the modulation of inflammatory cytokines and chemokines production (reviewed in [133]). Despite established roles in nonneuronal tissues, the expression and function of CCN family proteins in the CNS remain poorly understood.

### 5.1. CCN2/CTGF, Astrocytes, and Brain Insults

CCN2/CTGF is currently the only CCN family member in which expression has been demonstrated *in vivo* in CNS astrocytes. CCN2 expression has been detected not only in astrocyte somas and processes but also in a subpopulation of cortical neurons in adult rat brain, in tanycytes, and in the grey matter of the spinal cord [134]. CCN2 has been reported to be highly expressed in human astrocyte cell cultures [135] but was predominantly detected in neurons in healthy human brain and partially in subtypes of glial cells such as the glia limitans [136, 137].

Furthermore, CCN2 upregulation has been widely observed in reactive gliosis adjacent to the site of mechanical injuries caused by stab wound in rodents and also in the brain tissue of stroke patients [137] and following traumatic brain injury (TBI) [136]. Interestingly, in neurodegenerative diseases, CCN2 elevation has been detected both in neurons and reactive astrocytes. This dual expression was reported after excitotoxic damages provoked by kainic lesions in rat hippocampi [138] and in CNS tissue of multiple sclerosis (MS) [139], amyotrophic lateral sclerosis (ALS), [140] and AD [141] patients. In brains of AD patients, this increase was particularly significant around the amyloid plaques and neurofibrillary tangles [141]. CCN2 increase has also been highly correlated with glioblastoma (reviewed in [142]). Interestingly, CCN proteins are also proposed as potent modulators of cytokines and chemokines in different organs [133]. In primary cultured rat astrocytes, CCN2 mRNA levels were reported to be differentially modulated by distinct cytokines, showing an upregulation following treatment with TGF*β* and downregulation by TNF*α* [133]. Since both TGF*β* and TNF*α* are known to dramatically modify the glial and neuronal environment in lesioned tissue and neurodegenerative diseases [143–147], it is interesting to speculate that CCN2 production and release by reactive astrocytes may participate in the cascade of inflammatory events occurring in injuries and neurodegenerative processes. Finally, CCN2 has been demonstrated to bind to TrkA (neurotrophic tyrosine kinase receptor type 1) and p75NTR (p75 neurotrophin receptor), receptors which transduce neurotrophin signals [148]. How CCN2 elevation might influence neurotrophin signalling in brain diseases remains to be evaluated.

## 6. Perspectives: Astrocyte-Derived Matricellular Proteins as Tools to Reshape the ECM and Cell-Cell Interactions Following Injury and Disease

**Figure 1:**
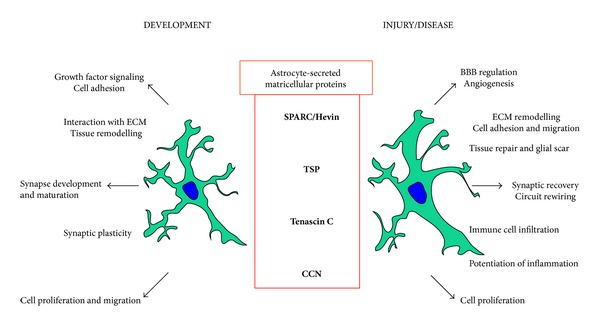
*Astrocyte-secreted matricellular proteins: Developmental tools for reactivity purposes*. Astrocyte-secreted matricellular proteins are highly expressed during the development of the CNS where they have multiple complex roles (left panel). In general, the expression of these molecules in the adult nervous system is reduced, but they are reexpressed at a high level in reactive astrocytes following injury or in disease states. Here, we summarise the possible roles of matricellular proteins secreted from reactive astrocytes (right panel).

Reactive astrocytes are found in nearly all situations following central nervous system injury and disease. Astrocyte reactivity is contextual [13] and leads to numerous morphological changes, such as an increase in the number and volume of processes [149], extension and elongation [150], and polarisation of processes around sites of injury [151]. This process requires dynamic modifications in cell-cell and cell-ECM interactions and involves the recruitment of other cell types, including immune cells [13]. Furthermore, the microenvironment surrounding the insult may modulate reactive astrogliosis and formation of the glial scar [152]. In this review, we have discussed several nonstructurally related families of matricellular proteins which are upregulated in reactive astrocytes. A common feature among these proteins is that they exhibit high levels of expression during development which is reduced to a lower level in the adult. However, their expression is reactivated following injury or disease. This pattern of expression is consistent with the fact that reactive astrocytes upregulate expression of proteins (GFAP, Vimentin, BLBP (brain lipid-binding protein), and Nestin) that are also present at high levels in radial glia or immature astrocytes [153]. Despite this, recent studies have shown that reactive astrocytes exhibit significant heterogeneity and the type of astrocyte reactivity occurring is dependent on the type and extent of CNS injury and disease [154–156]. For example, there is an increase in proliferating reactive astrocytes following lesions such as stroke but not in diseases such as Alzheimer's disease [129, 157]. Matricellular proteins have been described as dynamic regulators of the ECM and cell-cell interactions and are well placed to regulate the process of astrocyte reactivity and tissue remodelling occurring in the CNS following injury and in neurodegenerative disease (see Figure 1).

Ultimately, reactive astrogliosis can progress to form a glial scar, which has been shown to have both positive and negative effects on tissue recovery. The glial scar, which contains proliferating reactive astrocytes, forms a dense physical barrier around an injury. This protects the healthy brain from damaged tissue and inflammation and has been shown to play a role in repair of the BBB [14]. It is conceivable that matricellular proteins play an important role in modulating the glial scar, perhaps through regulation of glial cell proliferation and the surrounding vasculature. For example, SPARC is antiproliferator and can regulate microglia proliferation *in vitro* and *in vivo* following photothrombotic stroke [21, 45]. Conversely, TN-C was found to induce astrocyte proliferation *in vitro* [63]. In addition, SPARC, CCN2, and TSP1/2 have been implicated in angiogenesis and leukocyte infiltration in nonneuronal tissues [101, 158, 159] and therefore may serve to repair the BBB and restrain an immune response [160–162].

It has been well documented that the glial scar can inhibit axon regeneration; reactive “scar” astrocytes produce proteoglycans and other ECM molecules to inhibit axonal outgrowth, which ultimately limits functional recovery [163]. Through modulation of structural ECM components and adhesion and growth factor signaling (e.g., integrin signaling), astrocyte-secreted matricellular proteins may control axonal growth. In support of this, TN-C has been shown to both promote and inhibit axonal outgrowth and TSPs are known to positively affect growth [164]. All four matricellular protein families modulate cell adhesion *in vitro*, where they have been reported to restructure actin-containing focal adhesions and stress fibers [165]. This may translate into an *in vivo* role of “opening up” the ECM to create a permissive microenvironment for facilitating cell migration, outgrowth, angiogenesis, synaptic remodelling, and morphological plasticity. At the same time, upregulation of matricellular proteins may have negative consequences for tissue recovery and progression of disease. For example, matricellular proteins could promote an inflammatory response [19] and chronic inflammation is thought to be detrimental in neurodegenerative diseases such as Alzheimer's disease [166–168].

Huge challenges are awaited in order to understand the secretion of matricellular proteins from reactive astrocytes and functions in the CNS. Given their potential to modulate the microenvironment surrounding regions of brain injury and disease, astrocyte-secreted matricellular proteins may represent important therapeutic targets for CNS repair.
